# Childhood life events, immune activation and the development of mood and anxiety disorders: the TRAILS study

**DOI:** 10.1038/tp.2017.62

**Published:** 2017-05-02

**Authors:** I Jonker, J G M Rosmalen, R A Schoevers

**Affiliations:** 1 Department of Psychiatry, Interdisciplinary Center for Psychopathology and Emotion Regulation (ICPE), University of Groningen, University Medical Center Groningen, Groningen, The Netherlands; 2 Department of Internal Medicine, University of Groningen, University Medical Center Groningen, Groningen, The Netherlands

## Abstract

The experience of childhood life events is associated with higher vulnerability to develop psychiatric disorders. One of the pathways suggested to lead to this vulnerability is activation of the immune system. The aim of this study is to find out whether the association between childhood life events and the development of mood and anxiety disorders is predicted by the activation of the immune system. This study was performed in TRAILS, a large prospective population cohort, from which a subgroup was selected (*N*=1084, 54.3% female, mean age 19.0 (s.d., 0.6)). Childhood life events before age 16 were assessed using questionnaires at age 12, 14, 16 and 19. Immune activation was assessed at age 16 by elevated high-sensitive C-reactive protein (hsCRP) and by levels of immunoglobulin G antibodies against the herpes viruses herpes simplex virus 1, cytomegalovirus and Epstein–Barr virus. At age 19, the presence of mood and anxiety disorders was determined using the World Health Organization Composite International Diagnostic Interview Version 3.0. Regression analyses were used to study the association between life events, the inflammatory markers and mental health. We found that childhood life events score was associated with risk of mood disorders (*B*=0.269, *P*<0.001) and anxiety disorders (*B*=0.129, *P*<0.001). Childhood life events score was marginally associated with elevated hsCRP (*B*=0.076, *P*=0.006), but not with the antibody levels. This was especially due to separation trauma (*P*=0.015) and sexual abuse (*P*=0.019). Associations lost significance after correcting for lifestyle factors such as body mass index and substance abuse (*P*=0.042). None of the inflammatory markers were associated with development of anxiety disorders or mood disorders. In conclusion, the life event scores predicted the development of anxiety disorders and mood disorders at age 19. Life event scores were associated with elevated hsCRP, which was partly explained by lifestyle factors. Elevated hsCRP was not associated with the development of psychiatric disorders at age 19.

## Introduction

Childhood life events are associated with higher vulnerability to develop various psychiatric disorders, and the risk increases with the number of life events subjects have experienced.^1, 2^ Apart from psychological and social pathways, also several biological pathways have been suggested to play a role in this vulnerability. The experience of childhood life events is associated with hypothalamic-pituitary-adrenal axis dysregulation, different genetic and epigenetic influences on stress regulation, and with activation of the immune system.^3^ An activated immune system is associated with the presence of various psychiatric disorders. Depressive disorders,^4^ both poles of bipolar disorder,^5, 6^ dysthymic disorder,^7^ schizophrenia^8^ somatization^9^ and anxiety disorders in men^10^ all are found to be associated with an activated immune system. A recent meta-analysis found similar patterns of activation of the immune system in acute and chronic phases of schizophrenia, bipolar disorder and major depressive disorder. This finding makes it more likely that there is a common underlying pathway for immune disorders across these conditions.^11^ A possible explanation is that stress may cause activation of the immune system, which in turn could influence brain functioning and this could contribute to the development of psychiatric disorders. Immune activation has been found to influence brain functioning through several processes such as inhibiting neuroplasticity, activating the indoleamine 2,3 dioxygenase enzyme and thereby influencing the serotonin levels, and upregulating both dopamine and serotonin transporters.^12^ Activation of the immune system could therefore be a pathway linking traumatic or stressful experiences to later onset of psychopathology. Stressful experiences early in life could lead to a chronic state of immune activation, which would increase the vulnerability for the development of psychiatric disorders.

Studies towards activation of the immune system have focused on important pro-inflammatory cytokines, such as interleukin-6 (IL-6) and tumor necrosis factor alpha (TNF-α). C-reactive protein (CRP) is often used as a final common pathway, as this protein is generated in the liver in reaction to pro-inflammatory cytokines.^13^ Another approach to assess the functionality of the immune system is by measuring antibodies to common herpes viruses, such as herpes simplex virus type 1 and 2 (HSV1 and HSV2), Epstein–Barr virus (EBV) and cytomegalovirus (CMV). Many individuals have been infected by these viruses, and the cellular immune system keeps the virus inactive.^14^ It should be noted that stress-related low-grade immune activation, measured with markers such as high-sensitive-CRP (hsCRP), does not mean that the immune system works more effectively. Stress has been found to compromise the cellular immune system in such a way that reactivation of a latent virus can occur, as reflected in levels of virus-specific immunoglobulin G antibodies.^15, 16^ Reactivation of viruses can therefore be seen as a sign of impairment of the cellular immune system. Furthermore, earlier studies have shown an association between the presence of viral antibodies and the development of various psychiatric disorders. A systematic review on bipolar disorder found mixed results for perinatal infection with herpes viruses.^17^ A meta-analysis on schizophrenia found an association with presence of HSV 2 antibodies^18^ and a meta-analysis on depression found an association with presence of EBV and HSV1 antibodies.^19^

The association between an activated immune system and life events experienced in childhood has been studied in different age groups. Childhood life events were found to be associated with pro-inflammatory cytokines and CRP in elderly,^20, 21^ and in adults.^22, 23, 24^ However, the type of pro-inflammatory marker that is associated with the number of life events differed between the studies. For example, three studies in adults found an association between CRP and the number of traumatic childhood life events,^22, 24, 25^ but another study did not. This study, however, did find an association of traumatic childhood life events with TNF-α and IL-6.^23^ Similarly, antibody levels to HSV1, EBV and CMV in adults were also found to be associated with the number of childhood life events.^26, 27^ In adolescents, associations between life events and inflammatory markers have only been found under certain conditions. One study found associations between IL-6 and CRP, and the number of life events, which became non-significant when adding body mass index (BMI) as a covariate.^28^ Another study in adolescents found an association between childhood life events and IL-6 and CRP only in the group that developed depressive disorder 6 months later.^29^ A third study found an association between number of life events and CRP only when the adolescents had no positive engagement coping.^30^ Cellular immunity was also studied in adolescents, by measuring antibody levels to EBV. An association between antibody levels and number of life events was found in girls, but not in boys.^31^ An explanation for the difference in results could be related to the design of the previous studies. Most studies, especially those in elderly and adults, had a cross sectional design, thereby facing the problem of reverse causality and attention bias regarding the experience of life events. Another explanation for the difference in results could be related to the assessment of childhood trauma, which varied widely across the studies. However, most studies included verbal, sexual and physical abuse.^21, 27, 32^ Also, separation trauma, such as divorce of parents or death of a parent, is often included as a trauma in these studies.^20, 21, 22, 33, 34^ Recently meta-analyses have summarized the literature and found an association of adult inflammatory status, with the markers CRP, IL-6 and TNF-α, and exposure to childhood trauma.^35, 36^ Subgroup analyses for specific types of trauma (sexual, physical or emotional abuse and parental absence) revealed that these differentially impacted on inflammatory markers.^36^

Thus, previous studies on the association between the number of childhood life events and the activity of the immune system have involved samples of different ages. In adults, inconsistent associations were reported between childhood life events and various inflammatory markers. In adolescents, associations between life events and immune activation were only found in specific subgroups. Importantly, to date, no studies have prospectively assessed the link between childhood life events, the immune system and the development of various psychiatric disorders. Studying this association prospectively could give information on whether childhood life events may lead to a state of chronic low-grade immune activation and possible also impairment of the immune system. Ideally, such studies should be performed in adolescence. First of all, life events that take place in childhood are known risk factors for psychopathology that often develops during adolescence.^37^ A second advantage of studying adolescents is that, as life progresses, more and more factors such as ageing and medical illnesses may influence the immune system, complicating the interpretation of the association between childhood life events and immune system activation. Furthermore, there is less recall bias of childhood life events in adolescence, as less time passed after the events happened.

The aim of this study was to investigate whether activity of the immune system provides a pathway linking childhood life events with the development of mood and anxiety disorders. We hypothesized that the experience of life events is associated with the development of these psychiatric disorders at age 19, and that this association is predicted by an activated immune system. The second aim was to investigate whether this association differs for the different types of trauma.

We tested our hypotheses in the adolescent cohort TRAILS (TRacking Adolescents’ Individual Lives Survey), a large general population cohort from the north of the Netherlands, which follows children from the age of 11 with measurements of psychopathology and a comprehensive set of other potentially related variables every 2 years. We studied the association between life events before age 16, activity of the immune system at age 16, as measured by levels of hsCRP and immunoglobulin G antibodies to CMV, EBV and HSV1, and the development of mood and anxiety disorders at age 19.

## Materials and methods

### Study population

The study is part of TRAILS, which is a large prospective population study of 2230 Dutch adolescents from the north of the Netherlands examining causes and outcomes of physical and mental health from childhood into adulthood. The survey was approved by the national ethical committee ‘Centrale Commissie Mensgebonden Onderzoek’. Written informed consent was obtained from children and their parents or caretakers. A more elaborate description of the design can be found elsewhere.^37^ In short, all 135 primary schools in the five major municipalities in the northern part of the Netherlands were asked to participate in this study. Of the children of the schools that decided to participate, subjects with mental retardation, physical incapability or language problems were excluded. The study started in 2001 with the first wave of measurements, after that four follow-up measurements were done every 2 years. During the third assessment wave, all participants were asked to donate a blood sample for biomarker analyses (mean age=16.2 year, s.d.=0.6 years). After consent had been obtained from the parents and the children, blood samples were taken at the schools and delivered to the University Medical Center Groningen, where serum was extracted and stored at −80 °C until analysis. During the fourth follow-up assessment at the age of ~19 years, 84.2% (*n*=1584) of all participants agreed to also participate in an interview to assess mental disorders. We performed analysis on the group of participants of which we had both the blood samples at age 16 and information on mental health at age 19 (*n*=1084).

### Childhood life events

The type of life events that was studied in association with immune parameters varies widely. At wave 4, the experience of trauma before age 16 was assessed.^38, 39^ Four domains of trauma were assessed, sexual abuse, physical abuse, verbal abuse and separation trauma. To determine sexual abuse, the participants were asked whether an adult family member, friend of the family or stranger had ever, before the participant was 16, showed his/her genitals or masturbated in front of them; had sexually assaulted them; had forced them to touch him/her in a sexual manner; had attempted to have intercourse or had actually had intercourse with them. These five items on sexual abuse could be answered with ‘happened never’ (=0), ‘happened once’ (=1) or ‘happened more than once’ (=2). To determine physical abuse, participants were asked whether a parent or caretaker had ever, before the participant was 16, hit them with a belt, brush, stick or other hard object; had hit them with a fist or kicked them very hard; had shaken or pinched them; had beaten them up (that is, hit them in succession); or had threatened them with a knife or other weapon. To determine verbal abuse, participants were asked whether, before the age of 16, their parents shouted to them; parents threatened to hit them, parents called the participants stupid or lazy or similar things; parents threatened to send the participant away from home; parents cursed or scolded towards the participant. The five items on physical and the five items on verbal abuse could be answered with ‘never happened’ (=1), ‘happened once or twice’ (=2), ‘happened sometimes’ (=3), ‘happened often’ (=4) or ‘happened very often’ (=5). We chose to add information on separation trauma, taking together three items on separation, namely, parental death, parental divorce and out-of-home placement. Parental death or parental divorce was assessed at age 12, 14 and 16. Out-of-home placement was assessed by asking whether the children had been away from home for >3 months (wave 1), whether the participants had lived with another family (wave 2) and whether the participants were sent away from home (wave 3). Because of the different outcome scales for the various trauma types, we computed a *z*-score for each type of trauma, by assessing mean item scores per trauma type (verbal abuse, physical abuse, sexual abuse and separation trauma) and converting these into *z*-scores. We summed the *z*-scores of the domains to compute a total trauma score.

### Assessment of mental disorders

Mental disorders were assessed with the World Health Organization Composite International Diagnostic Interview (CIDI) Version 3.0,^40^ a fully structured lay-administered interview that generated diagnoses for 20 commonly occurring disorders, including mood disorders, anxiety disorders, behavior disorders and substance disorders. It assesses the presence of these diagnoses using both the criteria of the Diagnostic and Statistical Manual of Mental Disorders (DSM) IV and of the International Classification of Diseases 10. We chose to include in our analyses those psychiatric disorders previously found to be associated with inflammatory markers; this included mood disorders, comprising of bipolar disorder 1 and 2, dysthymia and major depressive disorder,^4, 5, 7^ and anxiety disorders,^10^ including panic disorder, generalized anxiety disorder, social phobia and obsessive compulsive disorder.^41^ For the assessment of mood and anxiety disorders, DSM-IV criteria were used. We based our analyses on diagnoses present in the previous year, as we tested whether inflammatory markers at age 16 would predict development of these disorders. We constructed a variable ‘life time diagnosis’, defined as having had a diagnosis earlier in life, but not in the previous year, to analyse the influence of a history of psychopathology. To exclude confounding of the longitudinal association by disorders at age 19, which were already present when inflammatory markers were assessed, we performed a sensitivity analysis excluding adolescents with anxiety or depressive disorders with an onset before age 17. As working with a categorical outcome reduces power and increases risk of false-negative results, we decided to repeat all analyses with continuous data, as a sensitivity analysis. We used the Adult Self-Report Questionnaire (ASR), which includes DSM-IV scale items of depression and anxiety.^42^

### Measurement of antibodies

All serological tests were carried out by standard procedure at the Stanley Laboratory of Developmental Neurovirology, Baltimore, MD, USA. To estimate viral activity, immunoglobulin G serum antibodies against agents of the herpes virus family (HSV1, CMV and EBV) were measured. For this measurement, a modified solid-enzyme immunoassay method was used, as was described before.^43, 44^ The immunoassays were performed by the reaction of the serum samples against the solid-phase antigen. The purified viral-envelope glycoprotein gG1 was used as the solid-phase agent for detecting HSV1.^45^ Reagents for the HSV1 assays were obtained from Focus Laboratories (Cypress, CA, USA; refEL0910G). The assays for the antibodies to CMV (catalog number IB19205) and EBV (catalog number IB79227) used antigens derived from virion proteins as previously described.^46^ Reagents for these assays were obtained from IBL-America (Minneapolis, MN, USA). After rinsing the serum samples that did not react to the antigens, the amount of antibody that was bound to the solid-phase antigen was quantified, by letting it react with enzyme-labeled anti-human immunoglobulin G and enzyme substrate. This process created a visual colored substrate, of which the optical density could be measured in a microplate colorimeter. Every assay was performed with our participant sample and a reference sample with low but detectable levels of antibodies against the target antigen. The amount of antibody in every sample was expressed in terms of the ratio of optical density of the test sample against that of the standard sample and was expressed in arbitrary units. For the first estimation of positivity, the sample of a participant was labeled as reactive when the ratio was at least 1.1. This cutoff point was based on a clinical trial of the assays of antibodies against HSV1.^47^ As a sensitivity analysis, we performed additional analyses with the continuous antibody levels in the total group, and with antibody levels in the seropositive groups using ratios of 0.5 and 2.0 to define reactivity. This did not yield different results.

### Measurement of hsCRP

CRP is produced by the liver in reaction to pro-inflammatory cytokines and is used as an indicator of systemic inflammation. hsCRP is more sensitive to small changes in systemic inflammation, as it is detected at lower levels.^48^ hsCRP was determined using a immunonephelometric method, BN2 of Siemens Medical Solutions USA (Malvern, PA, USA) with a lower detection limit of 0.175 mg l^−1^. Intra-assay coefficients of variance ranged from 2.1 to 4.4 mg l^−1^, and inter-assay coefficients of variation coefficients of variance ranged from 1.1 to 4.0 mg l^−1^. In our sample, hsCRP values were relatively low, as could be expected from a general sample of adolescents. Median value was 0.4 mg l^−1^, with 25th and 75th percentiles, respectively, 0.2 and 1.0 mg l^−1^. Thus, hsCRP values showed a skewed distribution, which did not improve upon transformation. Using continuous values of hsCRP would imply a substantial floor effect with a risk of false-negative results. For this reason, we decided to dichotomize the hsCRP value, with elevated hsCRP indicated by a cutoff of 2.0 mg l^−1^. In previous studies on inflammation in mood disorders, it was found that hsCRP levels >2.0 mg l^−1^ were relevant in mood disorders.^49, 50, 51^ Participants with an hsCRP value >10 mg l^−1^ were excluded from the analyses.

### Covariates

We included socioeconomic status (SES) as a covariate, as SES was found to be associated with inflammatory parameters as well as with childhood life events.^52^ A value for SES was estimated using five indicators: family income, educational level of the father and the mother, and occupational level of both parents using the International Standard Classification of Occupations.^53^ Factor analysis in this population showed a Crohnbach’s alpha of 0.84.^54^ As ethnicity is found to be associated with both life events and inflammatory marker levels,^25^ we also included ethnicity as a covariate, split into Dutch and non-Dutch. We included BMI as covariate, as it is associated with CRP and it was found to mediate the association between adverse events and CRP.^28^ At age 16, weight and height were measured and BMI was calculated. Furthermore, we included use of alcohol as a covariate, as this is associated with inflammatory status^55^ as well as the occurrence of various psychiatric disorders.^56^ The last covariate that we included was nicotine use, as this is associated with inflammatory status,^55^ childhood life events^57^ and psychiatric disorders.^58^ Both alcohol and nicotine use were based on self-reported use in the past 4 weeks (any use/no use).^59^ In the analyses in which we tested associations with psychopathology as outcome, we added ‘life time diagnosis’ as a covariate.

### Statistical analysis

Logistic regression analyses were used to study the association between life events and mental health. This was done for anxiety disorders as well as depressive disorders, with the life events score as independent variable and the presence of a mood disorder or the presence of an anxiety disorder as dependent variable. Subsequently, logistic regression was used to test whether the childhood life events score was associated with elevated hsCRP, with the life events score as independent variable, and hsCRP as dependent variable. These analysis were repeated for the different categories of life events. For the association of the childhood life events score with the antibody levels, we used multiple linear regression, with the life events score as independent variable, and the antibody levels as dependent variable. For each type of antibody, we performed the analyses with the continuous antibody levels, in the seropositive group. Because of non-normal distribution of data, we performed bootstrapping analyses in these multiple regression models. Next, logistic regression was used to test whether elevated hsCRP and viral antibodies are associated with the presence of psychiatric disorders, with hsCRP or viral antibodies as independent variables and the presence of any psychiatric disorder as dependent variable. When the life events score was found to be associated with inflammation, and inflammation was found associated with the psychiatric disorders, we planned to repeat the first model, testing the association between life events and psychiatric disorders, with hsCRP and/or the viral antibody levels added to the model as a mediator. In all analyses, two models were tested, the first adding personal factors as covariate, such as sex, ethnicity and socioeconomic status. The second model also included health behavior as covariate, including BMI, alcohol use and nicotine use. For our sensitivity analyses with continuous ASR scores as outcomes multiple linear regression models were used. We added a table of univariate associations with the outcomes using linear regression analyses.

## Results

### Sample characteristics

The study sample consisted of 1084 adolescents, with complete data on life events of 976 adolescents. Sample characteristics are summarized in Table 1. The prevalences of the disorders in the seropositive groups is represented in Table 2. The univariate associations of the covariates in our analysis with the outcomes was represented in Table 3.

### Life events and psychiatric disorders

Life events score before age 16 was associated with presence of mood disorders at age 19 (*B*=0.269, *P*<0.001, odds ratio (OR) 1.93, with a 95% confidence interval of 1.21–1.41) and anxiety disorders (*B*=0.129, *P*<0.001, OR 1.14, with a 95% confidence interval of 1.06–1.22). Sensitivity analyses with continuous outcome of ASR DSM-IV scales were also statistically significant, for depression scores (*B*=0.042, *P*=0.001) and anxiety scores (*B*=0.049, *P*=0.001).

### Life events and immune activation

We performed eight tests to assess the association between the inflammatory markers and the development of psychiatric disorders, using Bonferroni correction with the α level set at 0.006. The life events score was significantly associated with elevated hsCRP, but this association lost its significance after adjusting for BMI, nicotin use and alcohol use, as is shown in Table 4. We found no association of the life event score with levels of antibodies to EBV, CMV or HSV1 at age 16. Results are summarized in Table 5.

To find out which type of life event was mainly responsible for the association with elevated hsCRP, we repeated the analyses for various subgroups of life events. We found that, in our population, especially separation trauma and sexual abuse were associated with elevated hsCRP, but after Bonferroni correction these associations no longer reached statistical significance. The association between sexual abuse and elevated hsCRP did not remain significant after correcting for lifestyle factors. Results are summarized in Table 6.

As a sensitivity analysis, we repeated the analyses with the higher hsCRP levels included, and this diminished the association between life events and elevated hsCRP (full model: *B*, 0.06; s.e., 0.04; *P*=0.074, OR, 1.06; confidence interval 0.994–1.141; only adjusted for demographics: *B*, 0.08; s.e., 0.032; *P*=0.014, OR, 1.08; confidence interval 1.10–1.15).

### Immune activation and psychiatric disorders

We used eight tests to assess the association between the inflammatory markers and the development of psychiatric disorders. Using Bonferroni correction, we set the *α* level at 0.006. There was no significant association between hsCRP and the occurrence of the various psychiatric disorders. This was true for the model that adjusted for sex, ethnicity, SES and lifetime diagnoses (*B*=0.04, *P*=0.894, OR=1.04) and for the model that also adjusted for the lifestyle factors BMI, tobacco use and alcohol use (*B*=0.17, *P*=0.613, OR=1.19). There was also no association between the antibody levels and the occurrence of psychiatric disorders, as is summarized in Table 7. Analyses with ASR DSM-IV scales of depression and anxiety as outcome gave essentially the same results, as did analyses excluding adolescents with diagnoses with an onset before age 17.

As all four inflammatory parameters were not associated with the occurrence of depressive disorder or anxiety disorder, our results do not suggest that life events lead to these disorders through inflammation.

## Discussion

Our findings confirmed the first hypothesis that experience of life events before age 16 predicted the development of anxiety disorders and mood disorders at age 19. The total life event score was prospectively associated with elevated hsCRP.

This association lost significance after adjusting for health behaviors, although the estimate for the effect of life events on elevated hsCRP remained relatively unchanged. Of the types of life events, only physical abuse was not associated with elevated hsCRP. The association of elevated hsCRP with verbal abuse approached significance after correcting for multiple testing. Only the model including the sexual abuse score changed significantly after adjusting for health behaviors. Therefore, this study does not provide evidence that elevated hsCRP was associated with the development of psychiatric disorders at age 19.

A major strength of our study is its prospective design. The experience of traumas was assessed at age 19, which decreases the risk of recall bias. Another important advantage of performing this study in adolescents is that the effect of age-related factors on the immune system is limited. This reduces the risk that unknown confounders influenced our outcomes. However, there are also some limitations that need to be addressed. We did not exclude adolescents with externalizing psychiatric diagnoses from our control group, such as addictions, OD and attention deficit hyperactivity disorder. We chose mood disorders and anxiety disorders because these disorders have been found to be associated with inflammatory markers in the past. Attention deficit hyperactivity disorder is a diagnosis that by definition starts before age 12, meaning that immune activation at age 16 will not predict the development of this disorder at age 18. Also, OD is a disorder that is typically diagnosed before age 19. However, if an association between externalizing disorders and immune activation exists, it could have diluted the effects in our analyses. Using the CIDI to assess psychopathology contributes to the strength of our paper. The CIDI uses DSM criteria and therefore approaches the assessment of psychopathology as is done in the clinic. Furthermore, using CIDI makes our results comparable to studies in clinical samples. A disadvantage of working with a categorical outcome is a reduction in power and increase in risk of false-negative results. This is especially true as we combined diagnoses in a broader group. However, previous studies have found similar patterns of activation of the immune system in different psychiatric disorders, which makes it likely that there is a common underlying pathway for immune disorders across these conditions.^11^ For this reason, and because of the problem of false positives associated with multiple testing, we decided not to test for the possible associations between inflammation and disorders within the categories we tested. To explore the possible influence of our decision to use a categorical outcome, we performed sensitivity analyses in which categorical outcomes were replaced by continuous outcomes based on the ASR DSM-IV scales of depression and anxiety. We found essentially the same results as with categorical CIDI outcomes. This finding suggests that sub-threshold symptom scores for psychopathology were not associated with the immune markers that were measured in our sample. It makes it less likely that there was an association with the measured inflammatory markers and prodromes of mood or anxiety disorders.

Because of the timing of our assessments, we came across several limitations. First of all, we have no information on immune activation before age 16. Therefore, we have no information on when the low-grade inflammation started, and we cannot be sure that it really followed the experience of stressful life events. Second, we wanted to specifically study the development of psychiatric disorders after the measurement of inflammatory markers at age 16. Because of the timing of our measurements, it is possible that some of the adolescents that had a psychiatric disorder in the year before age 19, also had a psychiatric disorder at age 16, during or before the assessment of inflammatory markers. Adjustment for the presence of a lifetime diagnosis and sensitivity analyses excluding the adolescents with a diagnosis with an onset before the age of 17 revealed essentially the same results as in the whole population. Another potentially problematic group is adolescents who developed a psychiatric disorder after age 16, but already recovered before age 18. These adolescents were included in the healthy group, and will likely have weakened the association between inflammatory markers and psychopathology. Furthermore, some of our participants could develop psychiatric problems later in life, of which the underlying physiological and biological mechanisms were already present. Alternative explanations exist for the association between life events, inflammatory markers and the development of psychiatric disorders, which we could not focus on due to the timing of our assessments. Specifically, we could not focus on more direct effects of stress on the immune system. Furthermore, it is possible that it is not chronic activation but sensitization of the immune system that is responsible for the association between life events and psychopathology. Trauma and mitogen-induced cytokine production were previously found to be associated with increased sensitization to novel stressors,^60, 61, 62^ thereby making people at risk for the development of psychiatric disorders. Unfortunately, we were not able to study sensitization due to the absence of data on life stress between the age of 16 and 19.

An important limitation when studying the immune system is that we did not adjust our analyses for physical health or drug use at age 16. We tried to diminish the influence of severe physical problems associated with inflammation by excluding cases with high CRP levels, but the possibility of unmeasured confounding by physical health problems or drug use remains. We repeated analyses including participants with higher hsCRP levels, and this diminished the association between life events and hsCRP levels. This suggests that the higher hsCRP levels were not associated with life events scores, but probably had other causes. This confirms the hypothesis that stress leads to low-grade inflammation, rather than high inflammatory states. Apart from CRP, cytokines such as IL-6 and TNF-α have also been found to be associated with childhood life events and psychopathology.^35^ Unfortunately, we did not have data on these cytokines. Still, a recent meta-analysis suggests that among these markers, CRP is most consistently found to be associated with childhood life events.^36^The prevalence of the herpes viruses at age 16 in our population was low compared to findings in adolescents in age group 15–19 from other countries, with 70% seroprevalence for EBV in the USA,^63^ 1.6% for HSV2, 39% for HSV1 in the USA^64^ and 35% for CMV in Germany.^65^ Especially EBV seropositivity was very low in our cohort. Factors associated with EBV seropositivity include ethnicity, crowdedness, household income, household education level, and health insurance status^63^ and international variability in these factors might contribute to our low EBV seroprevalence.

We believe we were the first to prospectively study the associations between childhood life events, inflammatory markers, and mood and anxiety disorders 2 years later, in adolescents. Earlier studies did show an association between inflammatory markers and childhood life events,^22, 24, 25^ as well as psychiatric disorders.^4, 5, 7, 10^ We could replicate the finding that childhood life events were prospectively associated with elevated hsCRP, and that health behavior influences this association. Especially BMI influenced the association, with the strongest influence in the domain of sexual abuse. Importantly, however, we found no prospective association between hsCRP and the development of mood or anxiety disorders in our study. This finding fits in the context of the inconsistency of findings in earlier studies on the association of inflammatory markers with both childhood life events and psychiatric disorders.^34, 66^ Another study by Miller *et al.*^29^ combined childhood life events and inflammatory markers in the study of depressive disorder in adolescents. In subjects exposed to higher levels of childhood life events, the transition to depression was accompanied by increases in CRP and IL-6. This association between depression and inflammatory markers was not found in adolescents who did not have childhood adversities. There are two major differences in study design that could account for the differences in findings between this study and ours. The first is that, in the previous study, inflammatory markers were measured at the time of a current depressive episode. This could suggest that the activated immune system has a more direct effect, rather than a predisposing effect, as we hypothesized. Other studies have also shown that inflammatory markers, especially IL-6 and TNF-α, could vary according to different phases of psychiatric disorder.^67, 68^ The same was found for inflammatory monocyte gene expression; this was found to be significantly higher in patients with bipolar disorder then in healthy controls during a mood episode, but not in euthymic phase.^69^ These findings suggest that it is not activation of the immune system, but increased reactivity of the immune system that is associated with psychopathology. This could mean that immune reactivity in itself is a vulnerability factor for the development of psychopathology in relation to stress or trauma. Growing literature suggests that genetic variation (for example, in immune-related genes) may moderate the effects of childhood adversity. This immune reactivity could be explained by genetic factors and such individual differences in immune reactivity function could also explain the inconsistency of findings on effects of childhood trauma in current literature.^62^ However, a sensitization of the immune system by early life stress can also not be discarded. In that regard, it would have been interesting to study immune reactivity at the age of 16 in our sample, instead of the markers immune status that we had.

Another major difference in design between our study and the study by Miller *et al.*^29^ is that combined childhood life events and inflammatory markers in the development of depressive disorder was in diagnostic groups. We chose to combine several psychiatric classifications into two major diagnostic groups, whereas in the other study only major depressive disorder was studied. We did this to be able to study multiple psychiatric disorders, without having the problem of multiple testing. The disorders in the diagnostic groups we constructed have previously been associated with an activated immune system. However, we could have missed associations that were only present in specific diagnoses, but not in the diagnostic group as a whole. Especially when looking at current trends to study symptom dimensions instead of diagnostic groups, our approach of combining diagnoses could be seen as a limitation. A previous study in adults, for example, found that an association between metabolic parameters was only present in patients with an atypical subtype of depression.^70^ Another study found that metabolic and inflammatory dysregulation were associated with chronicity of depressive disorders.^71^ Such subtle differences would not be found in our data set with our variables being a sum of multiple disorders. Furthermore, reviews have concluded that there are differences in inflammatory markers between major depressive disorder and schizophrenia,^66^ and between bipolar disorder and schizophrenia.^67^ These findings suggest that even though several psychiatric disorders are associated with inflammatory dysregulation, the type of inflammatory response may differ per disorder. On the other hand, looking for associations in subgroups of disorders is a relatively new way of studying psychiatric disorders, and findings should be replicated before we know what these findings really mean.

We found no association between antibody levels and psychiatric disorders or life events, which could mean that adaptive immunity is not the part of the immune system that is mostly associated with stress and psychopathology. Antibody levels against herpes viruses have been studied only minimally in relation to life events,^34^ bipolar disorder^18^ and depressive disorder.^19^

Finally, we should consider the possibility that childhood life events do not lead to psychopathology via immune activation. It is possible that childhood life events and inflammatory markers are two different, independent etiological factors for the development of mood disorders.

In conclusion, we found that childhood life events were associated with elevated hsCRP, an association that could partly be explained by lifestyle factors. Childhood life events were also associated with the development of both mood disorders and anxiety disorders at age 19. However, this study does not provide evidence supporting the theory that childhood life events would contribute to the development of psychiatric disorders at age 19 via elevated hsCRP, 2 years before the development of these disorders. Our findings highlight the importance of life events in the development of psychiatric problems. They also confirm an association between childhood life events and hsCRP levels in adolescence. Repeating this study with a broader spectrum of inflammatory markers and with more frequent measurements of inflammation over time could give more information on whether immune activation does play a role in this association. It would also create the opportunity to study whether reactivity of the immune system might be the clue, rather than immune activation. Repeating the assessments later in the lives of the study participants could give more information on the development of psychiatric disorders.

## Figures and Tables

**Table 1:** Population characteristics

*General characteristics*	*% (*N*) or median (25th,75th)*
Age (years)	18.9 (18.5, 19.4)
Female sex	54.3 (590)

*Inflammatory status at age 16*	
hsCRP >2 mg l^−1^	12.3 (130)
HSV1 seropositivity	23.7 (258)
HSV1 antibodies (AU) in positive group	2.08 (1.68, 2.54)
EBV seropositivity	24.2 (263)
EBV antibodies (AU) in positive group	1.31 (1.19, 1.51)
CMV seropositivity	24.5 (266)
CMV antibodies (AU) in positive group	2.17 (1.95, 2.45)

*Psychiatric disorders in the previous year at age 19*	
Any mood disorder, including:	10.5 (112)

	N *(%) within mood disorders*
Bipolar I	3.6 (4)
Bipolar II	9.8 (11)
Major depressive disorder	84.8 (95)
Dysthymia	18.8 (21)

	N *(%) of total population*
1 disorder	7.5 (82)
2 disorders	2.8 (28)
3 disorders	0.2 (2)
Any anxiety disorder, including:	11.4 (124)

	N *(%) within anxiety disorders*
Generalized anxiety disorder	20.2 (25)
Panic disorder	8.9 (11)
Social phobia	62.9 (78)
Obsessive compulsive disorder	29.8 (37)

	N *(%) of total population*
1 disorder	9.1 (99)
2 disorders	2.1 (23)
3 disorders	0.2 (2)
Comorbid mood and anxiety disorder	3.4 (37%)

*Life events before age 16*	
Life events score	3.7 (3.2, 4.5)
Life events *z*-score	−0.4 (−1.7, 1.3)

*Covariates*	
Dutch ethnicity	90.5 (984)
SES low	16.9 (184)
SES average	48.4 (526)
SES high	33.9 (368)
BMI (kg/m^2^)	20.77 (19.24, 22.53)
Percentage that used nicotine in past 4 weeks	27.4 (298)
Percentage that used alcohol in past 4 weeks	75.1 (816)

Abbreviations: AU, arbitrary unit; BMI, body mass index; CMV, cytomegalovirus antibody levels; EBV, Epstein–Barr virus antibody levels; hsCRP, high-sensitive C-reactive protein; HSV1, herpes simplex virus type 1 antibody levels; SES, categorical measurement of socioeconomic status.

Life events score was computed by adding the mean scores on the various trauma types.

**Table 2:** Prevalence of psychiatric disorders in the different seropositive groups

	*Any depressive disorder (*n*)*	*Any anxiety disorder (*n*)*
HSV1 seropositivity (*n*=258)	26	25
EBV seropositivity (*n*=263)	32	20
CMV seropositivity (*n*=266)	29	30

Abbreviations: EBV, Epstein–Barr virus antibody levels; CMV, cytomegalovirus antibody levels; HSV1, herpes simplex virus type 1 antibody levels.

**Table 3:** Correlation table of covariates with the outcomes, regression analysis

	*Any mood disorder*	*Any anxiety disorder*	*Elevated hsCRP*	*HSV1*	*EBV*	*CMV*
Sex	−0.87^a^	−0.81^a^	−0.65^a^	0.21^a^	−0.04	−0.14
Ethnicity	0.53	0.22	0.44	0.04	−0.03	−0.04
SES	−0.22	−0.24	−0.22	0.06	0.05^a^	−0.01
BMI	0.05	0.02	0.19^a^	0.07	−0.00	−0.01
Alcohol	0.12	−0.39	0.39	-0.06	0.04	0.10
Nicotin	0.68^a^	0.29	0.69^a^	-0.10	0.05	−0.14

Abbreviations: BMI, body mass index; CMV, cytomegalovirus antibody levels in the seropositive group (*N*=266); EBV, Epstein–Barr virus antibody levels in the seropositive group (*N*=263); HSV1, herpes simplex virus type 1 antibody levels in the seropositive group (*N*=258); SES, socioeconomic status.

a Marks significant associations.

Regression coefficients (*B*) are presented.

**Table 4:** Association between life events score experienced before age 16 and elevated hsCRP at age 16 (*N*=946)

	*Elevated hsCRP*				
	*OR*	*95% CI*	B	*s.e.*	P*-value*
*Life events score*					
Unadjusted	1.11	1.03–1.16	0.1	0.03	**0.002**
Demographics^a^	1.1	1.03–1.17	0.09	0.03	**0.006**
Health behaviors^b^	1.08	1.00–1.16	0.08	0.04	0.042

Abbreviations: BMI, body mass index; CI, confidence interval; hsCRP, high-sensitive C-reactive protein; SES, socioeconomic status.

hsCRP tested in the whole sample (*N*=976); elevated hsCRP was defined with a cutoff 2.0 mg l^−1^. Life events score includes verbal, sexual or physical abuse and separation from parents, experienced before age 16. Bold faced <0.006.

a Adjusted for sex, ethnicity and SES.

b Adjusted for sex, ethnicity, SES, BMI, nicotine use and alcohol use.

**Table 5:** Association between life events score before age 16 and antibody levels at age 16

	B	*s.e.*	*95% CI*	P*-value*
*HSV1 levels (AU)*				
Life events score age 16				
Unadjusted	0.01	0.02	−0.08	0.788
Demographics^a^	0.01	0.02	−0.53	0.631
Health behaviors^b^	0.02	0.02	−0.09	0.39

*EBV levels (AU)*				
Life events score				
Unadjusted	0	0.02	−0.03	0.746
Demographics^a^	0	0.01	−0.03	0.627
Health behaviors^b^	0	0.01	−0.03	0.761

*CMV levels (AU)*				
Life events score				
Unadjusted	0	0.01	−0.05	0.864
Demographics^a^	0	0.01	−0.04	0.973
Health behaviors^b^	0	0.01	−0.05	0.81

Abbreviations: AU, arbitrary unit; BMI, body mass index; CI, confidence interval; CMV, cytomegalovirus antibody levels in the seropositive group (*N*=266); EBV, Epstein–Barr virus antibody levels in the seropositive group (*N*=263); HSV1, herpes simplex virus type 1 antibody levels in the seropositive group (*N*=258); SES, socioeconomic status.

a Adjusted for sex, ethnicity and SES.

b Adjusted for sex, ethnicity, SES, BMI, nicotine use and alcohol use.

95% CI is the confidence interval of bootstrapping. Life events score includes verbal, sexual or physical abuse and separation from parents, experienced before age 16.

**Table 6:** Association between the type of life events experienced before age 16 and elevated hsCRP at age 16

	*Elevated hsCRP >at age 16*				
	*OR*	*95% CI*	B	*s.e.*	P-value
*Verbal abuse score*					
Unadjusted	1.19	1.00–1.42	0.17	0.09	0.049
Demographics^a^	1.19	0.99–1.42	0.17	0.09	0.059
Health behaviors^b^	1.2	0.99–1.45	0.18	0.1	0.064

*Physical abuse score*					
Unadjusted	1.09	0.91–1.29	0.08	0.08	0.343
Demographics^a^	1.09	0.92–1.30	0.09	0.09	0.308
Health behaviors^b^	1.04	0.87–1.24	0.04	0.09	0.685

*Sexual abuse score*					
Unadjusted	1.22	1.07–1.40	0.2	0.07	0.004
Demographics^a^	1.18	1.03–1.36	0.17	0.07	0.019
Health behaviors^b^	1.11	0.95–1.31	0.11	0.08	0.198

*Separation trauma score*					
Unadjusted	1.27	1.07–1.52	0.24	0.09	0.008
Demographics^a^	1.25	1.05–1.50	0.23	0.09	0.015
Health behaviors^b^	1.26	1.04–1.53	0.23	0.1	0.02

Abbreviations: BMI, body mass index; CI, confidence interval; hsCRP, high-sensitive C-reactive protein; SES, socioeconomic status

.

a Adjusted for sex, ethnicity and SES.

b Adjusted for sex, ethnicity, SES, BMI, nicotine use and alcohol use.

hsCRP was tested in the sample of which we had data on life events (*N*=976); elevated hsCRP was defined with a cutoff of 2.0 mg l^−1^. The life events scores were obtained by calculating *z*-scores.

**Table 7:** The association between elevated hsCRP and herpes antibody levels at age 16 and the presence of any mood and any anxiety disorders at age 19

	*Any mood disorder*					*Any anxiety disorder*				
	*OR*	*95% CI*	B	*s.e.*	P	*OR*	*95% CI*	B	*s.e.*	P
*hsCRP>2 mg*/l										
Unadjusted	0.92	0.50–1.66	-0.10	0.30	0.773	1.46	0.76–2.80	0.38	0.33	0.251
Demographics^a^	1.04	0.57–1.92	0.04	0.31	0.894	1.70	0.85–3.40	0.53	0.36	0.136
Health behaviors^b^	1.19	0.61–2.30	0.17	0.34	0.613	1.68	0.86–3.26	0.52	0.34	0.129

*HSV1*										
Unadjusted	0.85	0.48–1.53	-0.16	0.30	0.589	1.07	0.61–1.87	0.07	0.29	0.816
Demographics^a^	0.98	0.51–1.86	-0.02	0.33	0.946	1.32	0.71–2.47	0.28	0.32	0.377
Health behaviors^b^	1.06	0.55–2.02	0.05	0.33	0.870	1.21	0.64–2.30	0.19	0.33	0.551

*EBV*										
Unadjusted	1.32	0.37–4.65	0.28	0.64	0.667	0.65	0.11–3.82	−0.43	0.91	0.632
Demographics^a^	1.33	0.36–4.98	0.29	0.67	0.669	0.60	0.10–3.73	−0.50	0.93	0.587
Health behaviors^b^	1.11	0.27–4.55	0.11	0.72	0.882	0.62	0.10–3.79	−0.48	0.93	0.602

*CMV*										
Unadjusted	0.58	0.26–1.27	−0.55	0.40	0.172	0.94	0.49–1.80	−0.07	0.34	0.842
Demographics^a^	0.52	0.22–1.22	−0.65	0.43	0.131	0.88	0.44–1.77	−0.12	0.36	0.728
Health behaviors^b^	0.63	0.28–1.43	−0.47	0.42	0.268	0.83	0.40–1.75	−0.18	0.38	0.630

Abbreviations: BMI, body mass index; CI, confidence interval; CMV, cytomegalovirus antibody levels in the seropositive group (*N*=266); EBV, Epstein–Barr virus antibody levels in the seropositive group (*N*=263); hsCRP, high-sensitive C-reactive protein tested in the whole sample (*N*=1084); HSV1, herpes simplex virus type 1 antibody levels in the seropositive group (*N*=258); SES, socioeconomic status.

a Adjusted for sex, ethnicity, SES and lifetime diagnosis.

b Adjusted for sex, ethnicity, SES, lifetime diagnosis, BMI, nicotine use and alcohol use.
